# NPC1 deficiency impairs cerebellar postnatal development of microglia and climbing fiber refinement in a mouse model of Niemann–Pick disease type C

**DOI:** 10.1242/dev.189019

**Published:** 2020-08-03

**Authors:** Bridget R. Boyle, Sierra E. Melli, Ruth S. Altreche, Zachary M. Padron, Fawad A. K. Yousufzai, Sarah Kim, Mariella D. Vasquez, Dawn M. Carone, Benjamin R. Carone, Ileana Soto

**Affiliations:** 1 Department of Molecular & Cellular Biosciences, Rowan University, Glassboro, NJ 08028, USA; 2 Swarthmore College, Department of Biology, Swarthmore, PA 19081, USA

**Keywords:** Cerebellum, CLEC7A, NPC, VGLUT2, Microglia, Pruning

## Abstract

Little is known about the effects of NPC1 deficiency in brain development and whether these effects contribute to neurodegeneration in Niemann–Pick disease type C (NPC). Degeneration of cerebellar Purkinje cells occurs at an earlier stage and to a greater extent in NPC; therefore, we analyzed the effect of NPC1 deficiency on microglia and on climbing fiber synaptic refinement during cerebellar postnatal development using the *Npc1^nmf164^* mouse. Our analysis revealed that NPC1 deficiency leads to early phenotypic changes in microglia that are not associated with an innate immune response. However, the lack of NPC1 in *Npc1^nmf164^* mice significantly affected the early development of microglia by delaying the radial migration, increasing the proliferation and impairing the differentiation of microglia precursor cells during postnatal development. Additionally, increased phagocytic activity of differentiating microglia was observed at the end of the second postnatal week in *Npc1^nmf164^* mice. Moreover, significant climbing fiber synaptic refinement deficits along with an increased engulfment of climbing fiber synaptic elements by microglia were found in *Npc1^nmf164^* mice, suggesting that profound developmental defects in microglia and synaptic connectivity might precede and predispose Purkinje cells to early neurodegeneration in NPC.

## Abstract (precis)

**Summary:** Genetic deficiency of *Npc1* impairs postnatal development of microglia and climbing fiber synaptic pruning in the mouse cerebellum.

## INTRODUCTION

Niemann–Pick disease type C (NPC) is a recessive genetic lysosomal storage disease caused by mutations in the NPC1 or NPC2 proteins, important transporters of cholesterol from endosomes and lysosomes (Patterson, 1993). Accumulation of cholesterol inside these intracellular organelles leads to progressive neurodegeneration, dementia and death in children. Developmental regression, ataxia, and cognitive impairment are found among the symptomatology of NPC. Although the average age of diagnosis is 10 years, 50% of NPC patients are diagnosed before age 7 and die before 12.5 years of age. However, the average age of death is 16 years, indicating that NPC onset and severity are variable but progressive (Garver et al., 2007). In fact, the nature and severity of neurological symptoms in NPC are directly correlated with the onset of the disease. Infantile manifestations of NPC include delay in motor milestones, gait problems, clumsiness and speech delay, whereas symptomatic manifestations of juvenile and adult onset of NPC include learning deficits, ataxia, dystonia and psychiatric symptoms (Mengel et al., 2013). Questions remain regarding how deficiency of the NPC1 protein perturbs developmental processes in the brain that could contribute to the early development of dementia and neurodegeneration.

Studies in mice and cats have revealed that Purkinje cells (PCs) are hypersensitive to loss of the NPC1 protein; in NPC these cells degenerate earliest and to a greater severity than other neurons in the brain (Vanier and Millat, 2003). This early degeneration of cerebellar PCs contributes to the development of early neurological symptoms, such as clumsiness, gait defects and ataxia, in both the human disease and animal models. Identifying the timeline and nature of pathological changes at the cellular and molecular level in the cerebellum caused by mutations in *Npc1* is crucial for understanding the mechanisms underlying the early dysfunction and degeneration of PCs. Interestingly, recent studies have shown that genetic inactivation of reactive microglia in *Npc1*^−/−^ mice reduces neurological impairment and increases their life span (Cougnoux et al., 2018). We recently found that engulfment and phagocytosis of dendrites by activated microglia occur early and precede PC loss in NPC (Kavetsky et al., 2019), demonstrating that activated microglia contribute to PC degeneration in NPC.

Although NPC is a childhood disorder, little is known about the effects of NPC1 deficiency on brain development. Moreover, how developmental deficits precede and contribute to neurodegeneration in NPC is not completely understood. Developmental delay in motor skill acquisition and significant reductions in synaptic and myelin proteins have been reported in the postnatal *Npc1^nmf164^* mouse (Caporali et al., 2016). In contrast to mice with complete deletion of the *Npc1* gene, *Npc1^nmf164^* mutant mice present a late onset and slower disease progression with severe motor deficits and neurodegeneration becoming evident at the young adult stage (Maue et al., 2012). Because degeneration of PCs and severe motor deficits are not found at early developmental stages, the *Npc1^nmf164^* mouse is an ideal model in which to study potential ‘silent’ cerebellar developmental defects and behavioral changes caused by NPC1 deficiency that precede the degeneration of PCs. In contrast to most common late-onset age-associated dementias, the early-childhood onset of neurological manifestations in NPC and other lysosomal storage disorders (LSDs) indicates potential disruption of neurodevelopmental processes. The impact of disrupted cholesterol trafficking by NPC1 deficiency in neural cells development is unknown, leading to a poor understanding of the origin of and preclinical mechanisms that lead to childhood dementia. Understanding the mechanisms by which NPC1 deficiency affects neural cells during development will not only expand our current knowledge of brain-behavior developmental processes, but will also provide potential therapeutic avenues to identify and delay the progression of NPC and other childhood dementias (Ford et al., 1951; Shapiro, 1994).

Previous work in our laboratory showed significant changes in microglia number, morphology and phagosome content in the cerebellum of *Npc1^nmf164^* mice at post-weaning age and before PC degeneration (Kavetsky et al., 2019). Interestingly, the migration, proliferation and differentiation of cerebellar microglia occur postnatally along with the development and differentiation of cerebellar neurons. In the brain, microglia play important roles during normal development, including the clearance of apoptotic cells and the elimination of redundant synapses. Importantly, compared with microglia from other regions of the brain, normal cerebellar microglia strongly display cellular and gene expression patterns that are commonly associated with cell clearance (Ayata et al., 2018), suggesting that cerebellar microglia are more phagocytic. To determine the impact of NPC1 deficiency in cerebellar microglia during postnatal development, different stages of microglia development, such as migration, proliferation and differentiation, were examined at the corresponding postnatal age. The findings of this study suggest that NPC1 deficiency not only affects the different phases of microglia development, but also increases phagocytosis activity in these cells, which promotes and amplifies profound synaptic defects during the postnatal development of the cerebellum.

## RESULTS

### Early changes in microglia are not the result of an innate immune response in the cerebellum of post-weaning *Npc1^nmf164^* mice

**Fig. 1.**
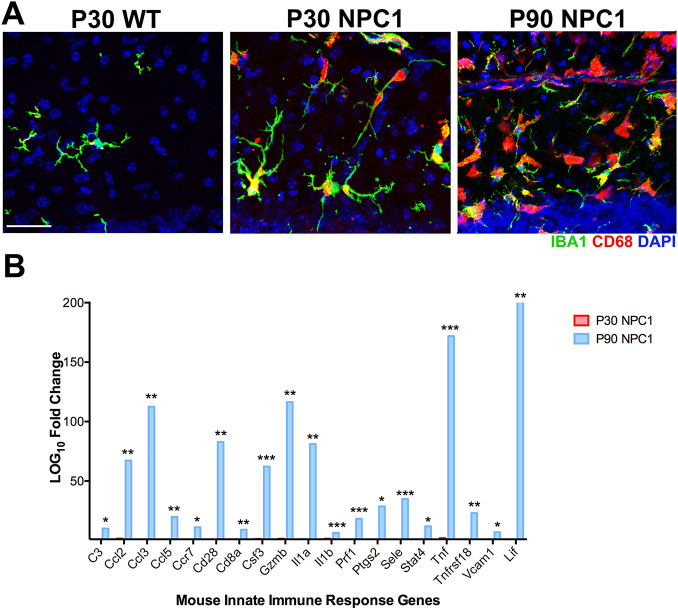
**Changes in innate immune responses are not evident in the cerebellum at post-weaning age in *Npc1^nmf164^* mice.** (A) Images from P30 and P90 mice showing IBA1^+^ microglia cells containing CD68^+^ phagosomes at the ML. (B) Expression changes of genes associated with the innate immune response comparing *Npc1^nmf164^* mice at P30 (red, *n*=3) and P90 (blue, *n*=3) versus WT mice (*n*=4). *P<0.05, **P<0.01, ***P<0.001. Scale bar: 30 µm.

As changes in *Npc1^nmf164^* microglia are evident at postnatal day (P) 30 (Kavetsky et al., 2019; Fig. 1A), we proceeded to test whether genes associated with an innate immune response were upregulated in the cerebellum of P30 *Npc1^nmf164^* mice. A PCR array plate containing primers for ∼92 genes associated with the mouse innate-immune response was used. RNA from the cerebellum of P90 *Npc1^nmf164^* mice was used as a positive control because at this age a severe loss of PCs is detected and the changes in microglia morphology and proliferation are more remarkable than at P30 (Fig. 1A) (Kavetsky et al., 2019). As expected, several genes, including cytokines (*Lif*, *Tnf*, *Csf3*, *Il1a*, *Ccl2* and *Ccl3*), endothelial-inflammatory genes (*Sele* and *Vcam1*), T-cell associated genes (*Gzmb*, *Cd3e*, *Cd28*, *Stat4*, *Prf1* and *Tnfrsf18*) and other proinflammatory molecules (*C3* and *Ptgs2*), were significantly increased (>5-fold) in the cerebellum of P90 *Npc1^nmf164^* mice compared with wild-type (WT) mice (Fig. 1B). However, no significant expression changes were found in the cerebellum of P30 *Npc1^nmf164^* mice compared with WT mice (Fig. 1B), suggesting that microglia changes at that early age are not the consequence of an immune or inflammatory response in *Npc1^nmf164^* mice. To determine whether microglia changes at P30 are associated with the effects of NPC1 deficiency on microglia development, a comprehensive analysis of cerebellar and microglia postnatal development was performed.

### Early radial migration of microglial precursors into the developing cerebellar cortex is reduced in *Npc1^nmf164^* mice

**Fig. 2.**
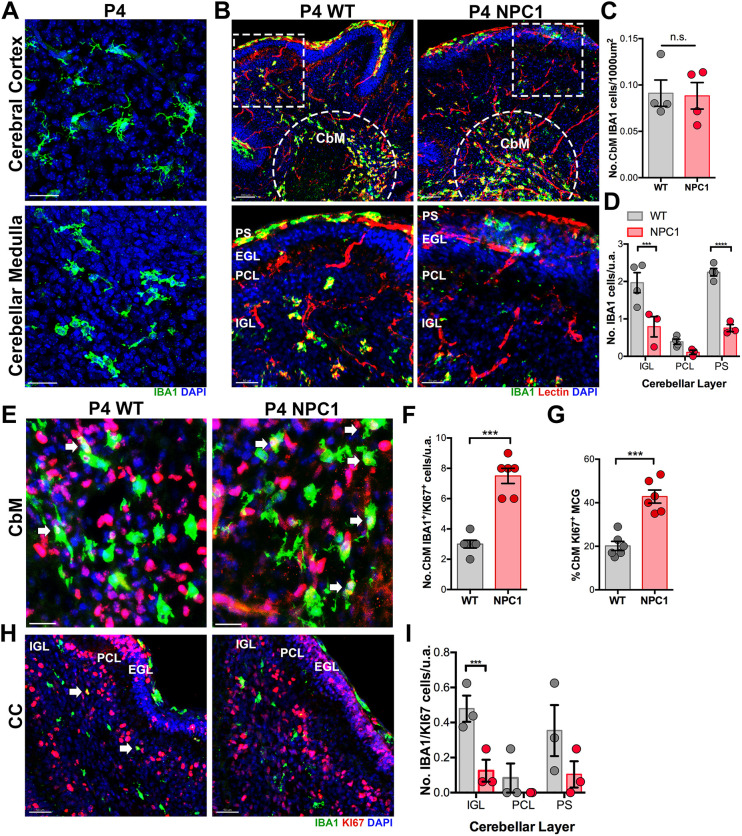
**Decreased radial migration of microglial precursors in P4 *Npc1^nmf164^* mice.** (A) IBA1^+^ differentiating microglia in the cortex versus IBA1^+^ round/ameboid microglial precursors in the cerebellum at P4 in WT mice. (B) IBA1^+^ microglial precursors concentrated in the developing CbM of the cerebellum (dashed semicircle). IBA1^+^ microglial precursors are observed migrating radially toward the cerebellar cortex layers (IGL, PCL and EGL) following Tomato Lectin^+^ capillaries. Boxed areas are shown at higher magnification below. (C) Quantification of IBA1^+^ cells in the developing CbM at P4. (D) Quantification of the number of IBA1^+^ cells in the IGL and PS at P4. (E) IBA1^+^ and KI67^+^ cells in the developing CbM at P4. (F) The number of IBA1^+^/KI67^+^ cells is significantly higher in the CbM of *Npc1^nmf164^* mice. (G) The percentage of KI67^+^ microglia (MCG) is significantly higher in the CbM of *Npc1^nmf164^* mice. (H) IBA1^+^ and KI67^+^ cells in the developing cerebellar cortex (CC) at P4. Arrows indicate IBA1^+^ cells that are KI67^+^. (I) The number of IBA1^+^/KI67^+^ cells is significantly lower in the IGL of *Npc1^nmf164^* mice. Data are presented as mean±s.e.m. *n*=3-4 mice. ***P<0.001, ****P<0.0001. n.s., not significant; u.a., unit area. Scale bars: 50 µm (A,E,H); 100 µm (B, top row); 50 µm (B, bottom row).

In the mouse, most cerebellar development occurs postnatally. As expected, at early postnatal stages such as P4, only round and ameboid IBA1^+^ microglial precursors were observed in the developing cerebellar medulla (CbM) whereas ramifying microglia were already found at this stage in the cerebral cortex (Fig. 2A). As described by others (Ashwell, 1990; Cuadros et al., 1997; Mosser et al., 2017), in the P4 WT cerebellum microglial precursors were concentrated in the developing CbM and were migrating tangentially towards the primitive cerebellar folia following tomato lectin^+^ blood vessels (Fig. 2B). Also, at this early postnatal stage, abundant microglial precursors were observed at the pial surface (PS) of the meninges (Fig. 2B). The beginning of the radial migration of microglial precursors into the different regions of the cerebellar cortex, including the inner granule layer (IGL), Purkinje cell layer (PCL) and external granule layer (EGL), is expected to occur at this early stage. Quantitative analysis of the density of IBA1^+^ microglial precursors in the CbM revealed no differences between P4 WT and *Npc1^nmf164^* mice (Fig. 2B,C). However, a significant reduction in the number of IBA1^+^ microglial precursors reaching the IGL and circulating the PS was found in *Npc1^nmf164^* mice compared with WT mice (Fig. 2B,D). At this early postnatal stage, very few IBA1^+^ microglial precursors had reached the PCL, and the EGL was completely devoid of them, as reported by others (Cuadros et al., 1997; Nakayama et al., 2018). No changes in vascularization (tomato lectin^+^ blood vessels) were observed between the P4 WT and *Npc1^nmf164^* mice (Fig. 2B, Fig. S1).

Proliferative activity in CbM microglial precursors was detected in both P4 WT and *Npc1^nmf164^* mice, but a significantly higher number and percentage of these CbM KI67^+^/IBA1^+^ microglial precursors were found in *Npc1^nmf164^* mice (Fig. 2E-G). Meanwhile, proliferative activity in microglial precursors at the IGL, PCL and PS was very low in P4 WT and *Npc1^nmf164^* mice, but a further significant reduction in proliferative microglial precursors was found in *Npc1^nmf164^* mice (Fig. 2H,I), mostly because of the delayed migration of these cells in the *Npc1^nmf164^* mice. Overall, these results suggest that NPC1 deficiency affects the ability of microglial precursors to migrate radially while increasing their proliferation during early postnatal cerebellar development.

### Increased density of precursor and maturing microglia in the developing cerebellum of P10 and P14 *Npc1^nmf164^* mice

**Fig. 3.**
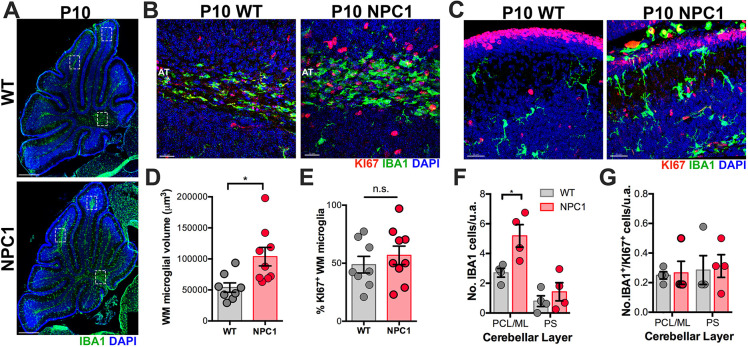
**Increased number of microglial precursors and differentiating microglia in *Npc1^nmf164^* mice at P10.** (A) Images of P10 whole cerebellum sections immunostained with IBA1 showing the distribution and abundance of IBA1^+^ cells in WT and *Npc1^nmf164^* mice. Boxes indicate some regions of WM. (B) High magnification images of the WMR immunostained with IBA1 and KI67 showing higher number of IBA1^+^ cells in the *Npc1^nmf164^* mouse. AT, axonal tract. (C) High magnified images in a region of the cerebellar cortex immunostained with IBA1 and KI67 showing higher number of IBA1^+^ cells in the *Npc1^nmf164^* mouse. (D) Quantification of the volume of IBA1^+^ microglia clusters in the cerebellar WMR. (E) No differences were found in the percentage of WM IBA1^+^/KI67^+^ cells between WT and *Npc1^nmf164^* mice. (F) Quantification of the number of IBA1^+^ cells in the PCL/ML and PS. (G) Quantification of the number of IBA1^+^/KI67^+^ cells in the PCL/ML and PS. Data are presented as mean±s.e.m. (D,E) *n*=2 images per mouse, *n*=4 mice; (F,G) *n*=4 mice. *P<0.05. n.s., not significant; u.a., unit area. Scale bars: 250 µm (A); 30 µm (B,C).

**Fig. 4.**
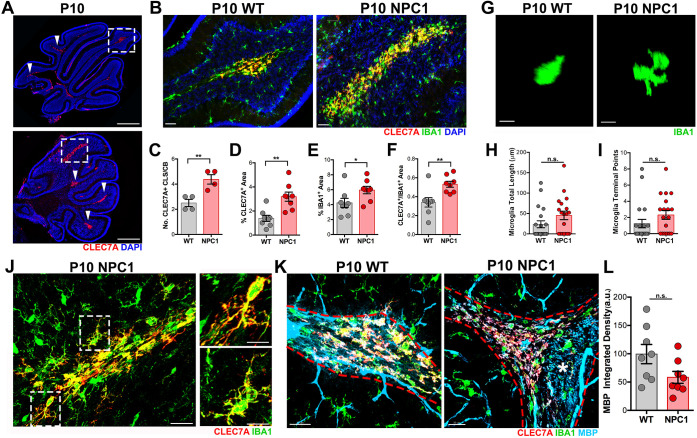
**Proliferative-region-associated microglia (PAM) are increased in the cerebellar WMR of P10 *Npc1^nmf164^* mice.** (A) Images of P10 whole cerebellum sections immunostained with CLEC7A showing the distribution and abundance of CLEC7A^+^ cells in WT and *Npc1^nmf164^* mice. (B) High magnification images of the boxed areas in A showing CLEC7A^+^ cells colocalizing with IBA1 in the cerebellar WMR of WT and *Npc1^nmf164^* mice. (C) Quantification of the number of CLEC7A^+^ clusters (CLS, arrowheads in A) per cerebellum (CB) in WT and *Npc1^nmf164^* mice. (D) Quantification of the percentage area immunostained by CLEC7A in WT and *Npc1^nmf164^* mice. (E) Quantification of the percentage area immunostained by IBA1 in WT and *Npc1^nmf164^* mice. (F) Fraction of the IBA1^+^ area also immunostained by CLEC7A^+^ in WT and *Npc1^nmf164^* mice. (G) Images of the morphology of P10 IBA1^+^ microglia that are CLEC7A^+^ in WT and *Npc1^nmf164^* mice. (H,I) Quantitative analysis of the total length (H) and number of terminal points (I) of P10 CLEC7A^+^ microglia. (J) Evidence of CLEC7A^+^ differentiating microglia in P10 *Npc1^nmf164^* mice. Boxed areas are shown at higher magnification on the right. (K) Immunostaining of WM track (dashed red line) with MBP showing myelinated axons intermingling between CLEC7A^+^/IBA1^+^ microglia in WT and *Npc1^nmf164^* mice. White asterisk indicates WM region with high levels of MBP. (L) Differences in MBP integrated density were not statistically significant between P10 WT and *Npc1^nmf164^* mice. Data are presented as mean±s.e.m. (C-F,L) *n*=2 images per mouse *n*=4 mice; (H,I) 19-20 cells per mouse, *n*=4 mice. *P<0.05, **P<0.01. a.u., arbitrary units; n.s., not significant; u.a., unit area. Scale bars: 250 µm (A); 50 µm (B); 10 µm (G); 30 µm (J,K).

Studies have shown that the proliferative activity of microglial precursors in the developing cerebellar CbM and folias white matter region (WMR) starts at birth and peaks at P7 in normal mice (Ashwell, 1990; Li et al., 2019). In fact, an abundant subpopulation of microglia in the WMR region was recently identified as proliferative region-associated microglia (PAM) (Li et al., 2019). When we examined the cerebellum of P10 WT and *Npc1^nmf164^* mice, we found that the volume occupied by IBA1^+^ microglia in the WMR (axonal tracts) was significantly higher in *Npc1^nmf164^* mice than in WT mice (Fig. 3A,B,D). However, the percentage of proliferative microglia in this region, as assessed by KI67 immunostaining, was similar between WT and *Npc1^nmf164^* mice (Fig. 3B,E). Similarly, the density of IBA1^+^ microglia in the PCL and molecular layer (ML) was significantly increased in *Npc1^nmf164^* mice without significant changes in KI67 immunoreactivity compared with WT mice (Fig. 3C,F,G). In addition, microglia in the cerebellar cortex of WT and *Npc1^nmf164^* mice were evidently maturing and ramifying (Fig. 3C), whereas the microglia in the WMR were ameboid shape and less ramified, an indication of a more immature cell (Fig. 3B). Because no differences in KI67 immunoreactivity at the cerebellar WMR were observed between P10 WT and *Npc1^nmf164^* mice, our results suggest that an increased number of microglia was produced in the cerebellar WMR of *Npc1^nmf164^* mice prior to the P10 stage (P4-P7), as reported by others (Li et al., 2019). To test this possibility, P10 WT and *Npc1^nmf164^* cerebellar slices were immunostained with CLEC7A (Fig. 4), a specific marker for PAM cells, the proliferation rate of which in WT mice is significantly reduced after peaking at P7 (Li et al., 2019). Interestingly, a significant higher number of CLEC7A^+^ clusters were observed in *Npc1^nmf164^* developing WMRs (Fig. 4A,C). Also, the area of these clusters of CLEC7A^+^ (Fig. 4D) and IBA1^+^ cells (Fig. 4E), as well as the fraction of the IBA1^+^ area that was CLEC7A^+^ (Fig. 4F), were significantly larger in *Npc1^nmf164^* mice compared with WT. The majority of P10 CLEC7A^+^ microglial precursors lacked processes and were primarily ameboid shape in both WT and *Npc1^nmf164^* mice (Fig. 4G-I). Although CLEC7A expression is absent in differentiated microglia (Li et al., 2019), we found a higher tendency of CLEC7A^+^/IBA1^+^ microglial precursors to have more terminal points in *Npc1^nmf164^* mice (Fig. 4H,I), and some CLEC7A^+^ differentiating microglia were observed in *Npc1^nmf164^* mice (Fig. 4J). Finally, because CLEC7A^+^/IBA1^+^ microglia actively phagocytose oligodendrocyte progenitor cells (OPCs) at P7 (Li et al., 2019), and decreased levels of myelin basic protein (MBP) have been found in P15 *Npc1^nmf164^* mice (Caporali et al., 2016), analysis of MBP immunostained cerebella was performed specifically in the regions where CLEC7A^+^/IBA1^+^ cells were located (Fig. 4K; blood vessels staining is artifactual). We found that MBP intensity inside the CLEC7A^+^ clusters tended to be decreased in *Npc1^nmf164^* mice, but due to variability in WT mice the result was not statistically significant (Fig. 4L).

**Fig. 5.**
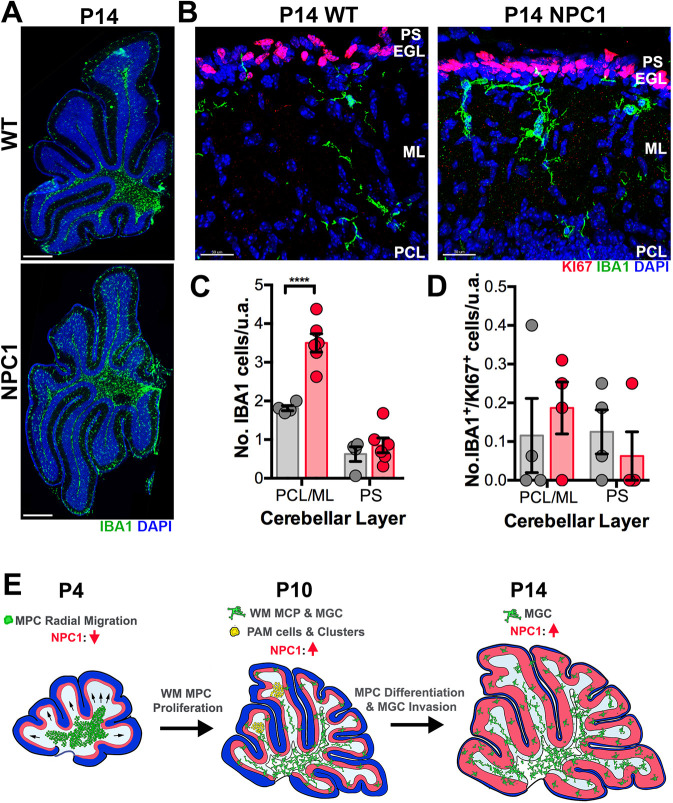
**Increased number of differentiating microglia in *Npc1^nmf164^* mice at P14.** (A) Images of P14 whole cerebellum sections immunostained with IBA1 showing the distribution and abundance of IBA1^+^ cells in WT and *Npc1^nmf164^* mice. (B) High magnification images of the cerebellar cortex (PCL, ML, EGL and PS) immunostained with IBA1 and KI67 showing higher number of IBA1^+^ cells in the *Npc1^nmf164^* mouse. (C) Quantification of the number of IBA1^+^ cells in the PCL/ML and PS at P14. (D) Quantification of the number of IBA1^+^/KI67^+^ cells in the PCL/ML and PS at P14. (E) NPC1 deficiency affects migration and proliferation events of microglial precursors (MPC), PAM and differentiating microglia (MGC) in the postnatal cerebellum. Data are presented as mean±s.e.m. (C) *n*=4 (WT) and 6 (*Npc1^nmf164^*) mice; (D) *n*=4 mice. ****P<0.0001. n.s., not significant; u.a., unit area. Scale bars: 250 µm (A); 30 µm (B).

At P14, the density of IBA1^+^ microglia was also significantly higher in the PCL/ML of *Npc1^nmf164^* mice compared with WT mice (Fig. 5A-C). However, the levels of IBA1^+^/KI67^+^ cells in P14 WT and *Npc1^nmf164^* mice were very low in the cerebellar cortex layers (Fig. 5B,D). Overall, our results suggest that the increased proliferation of WMR microglial precursors in *Npc1^nmf164^* mice leads to an increased density of microglia in the cerebellar cortex region as no increased microglia proliferative activity is detected in the cerebellar cortex at P10 and P14 (Fig. 5E).

### NPC1 deficiency affects microglia differentiation and ramification

**Fig. 6.**
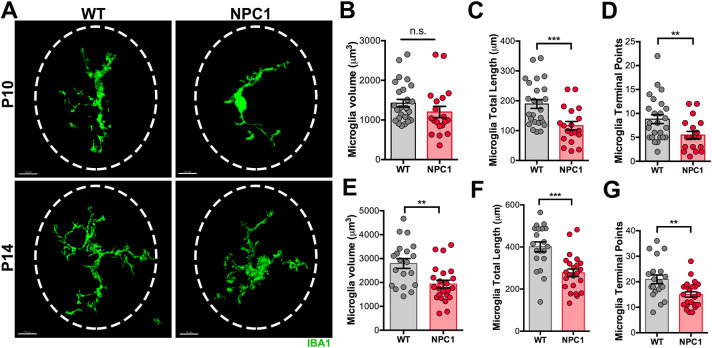
**Microglia differentiation is impaired in *Npc1^nmf164^* mice.** (A) Representative images of IBA1 microglia from P10 and P14 WT and *Npc1^nmf164^* mice show microglia that are less ramified and have shorter processes in *Npc1^nmf164^* mice. (B) Quantitative analysis of cell volume at P10. (C,D) Total length (C) and number of terminal points (D) of microglia processes at P10. (E-G) Volume (E), total length (F) and number of terminal points (G) of microglia processes at P14. Data are presented as mean±s.e.m. (B-D) *n*=25 cells (WT) from 4 mice, *n*=19 cells (*Npc1^nmf164^*) from 4 mice; (E-G) *n*=20 cells (WT) from 4 mice, *n*=23 cells (*Npc1^nmf164^*) from 4 mice. **P<0.01, ***P<0.001. n.s., not significant. Scale bars: 10 µm.

In the early days of cerebellar postnatal development, microglial precursors in the WMR were distinctly recognized by their round or ameboid shape (Fig. 2A,B). It was also evident that as these cells migrate to the cerebellar cortex, where PC dendrites and synaptic connections are developing, they begin to differentiate, ramify and extend their processes through the ML where neuronal synaptic connections are found (Fig. 3C). To determine whether NPC1 deficiency alters microglia differentiation, a quantitative analysis of IBA1^+^ cells morphology was performed in P10 and P14 WT and *Npc1^nmf164^* mice, using the ‘Filament Tracer’ tool (Imaris). Differences in microglia volume were not detected between WT and *Npc1^nmf164^* mice at P10 (Fig. 6A,B); however, at this stage, the microglia total length and the number of terminal points were significantly reduced in *Npc1^nmf164^* mice (Fig. 6C,D). At P14, the microglia volume, total length and terminal points were significantly lower in *Npc1^nmf164^* mice compared with WT mice (Fig. 6A,E-G). These results show that microglia in *Npc1^nmf164^* mice are less ramified and have shorter processes, suggesting that NPC1 deficiency impairs microglia differentiation and ramification during postnatal development.

### Phagocytic activity is increased in developing *Npc1^nmf164^* microglia

**Fig. 7.**
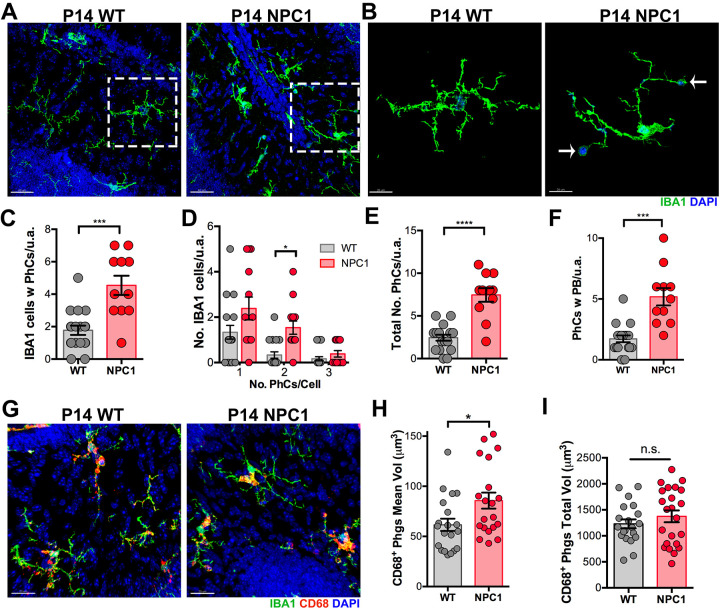
**Increased number of phagocytic cups in *Npc1^nmf164^* mice at P14.** (A) Images of P14 cerebellar sections at the ML showing the IBA1^+^ cells in WT and *Npc1^nmf164^* mice containing phagocytic cups. (B) High magnification images of the boxed areas in A showing *Npc1^nmf164^* microglia with two phagocytic cups containing pyknotic bodies (arrows). (C) Quantification of the number of IBA1^+^ cells with phagocytic cups (PhCs) in the ML of P14 mice. (D) Quantification of the number of IBA1^+^ cells with one, two or three phagocytic cups in WT and *Npc1^nmf164^* mice. (E) The total number of phagocytic cups per unit area in the ML. (F) The number of phagocytic cups containing pyknotic bodies (PB). (G) Images showing P14 microglia immunostained with IBA1 and CD68 in WT and *Npc1^nmf164^* mice. (H) Quantification of the mean volume of CD68^+^ phagosomes (Phgs). (I) Total (summed) volume of CD68^+^ phagosomes between WT and *Npc1^nmf164^* mice. Data are presented as mean±s.e.m. (C-F) WT *n*=13 images from 4 mice, *Npc1^nmf164^ n*=11 images from 4 mice. *P<0.05, ***P<0.001, ****P<0.0001. n.s., not significant; u.a., unit area. Scale bars: 40 µm (A); 20 µm (B,G).

Microglia play an important role in the clearance of apoptotic cells during development (Mosser et al., 2017). While analyzing microglia morphology in P14 postnatal mice, we noticed an abundant number of maturing microglia in the ML containing phagocytic cups, especially in *Npc1^nmf164^* mice. Phagocytic cups are cup-shaped endocytic vacuolar structures in ramified microglia that are formed by the ingestion of particles or cells during phagocytosis (Swanson, 2008). The number of microglia with phagocytic cups was significantly higher in *Npc1^nmf164^* mice compared with WT mice (Fig. 7A-C). We also found that microglia with at least two phagocytic cups were more abundant in the ML of *Npc1^nmf164^* mice, and we observed more phagocytic cups per image area (Fig. 7B,D,E). Interestingly, the number of phagocytic cups containing pyknotic bodies in the ML was higher in *Npc1^nmf164^* mice than in WT mice, suggesting that NPC1 deficiency increases microglial phagocytic activity in the developing cerebellum. Immunostaining of microglia with the CD68 antibody, a phagosome marker, showed that P14 WT and *Npc1^nmf164^* microglia were actively phagocytosing at this developmental stage, as CD68^+^ phagosomes were abundant in microglia from both mouse strains. Markedly, WT IBA1^+^ microglia at P14 had many small CD68^+^ phagosomes distributed through the cell body and processes (Fig. 7G), whereas in the *Npc1^nmf164^* microglia the majority of the CD68^+^ phagosomes were accumulated in the cell body (Fig. 7G). Quantitative analysis showed that the mean volume of CD68^+^ phagosomes per microglia at P14 was larger in *Npc1^nmf164^* mice than in WT mice (Fig. 7G,H); however, no differences were found in the total volume of CD68 between WT and *Npc1^nmf164^* microglia at this stage (Fig. 7I). CLEC7A expression is reactivated specifically in actively phagocytic disease-associated microglia (DAM) (Krasemann et al., 2017); therefore, to test whether P14 phagocytic cells are similar to DAM, P14 and P60 (NPC neurodegeneration stage) WT and *Npc1^nmf164^* cerebella were immunostained with CLEC7A. We found that CLEC7A^+^ microglia only reappear in the ML of *Npc1^nmf164^* mice during neurodegeneration at P60 (Fig. S2), and not at P14. Our results suggest that postnatal changes in NPC microglia are developmental alterations caused by NPC1 deficiency and not an immunological response. These results indicate that NPC1 deficiency alters the phagocytic activity and the distribution of phagosomes in developing cerebellar microglia.

### Microglia function in climbing fiber synapse elimination is altered in *Npc1^nmf164^* mice

**Fig. 8.**
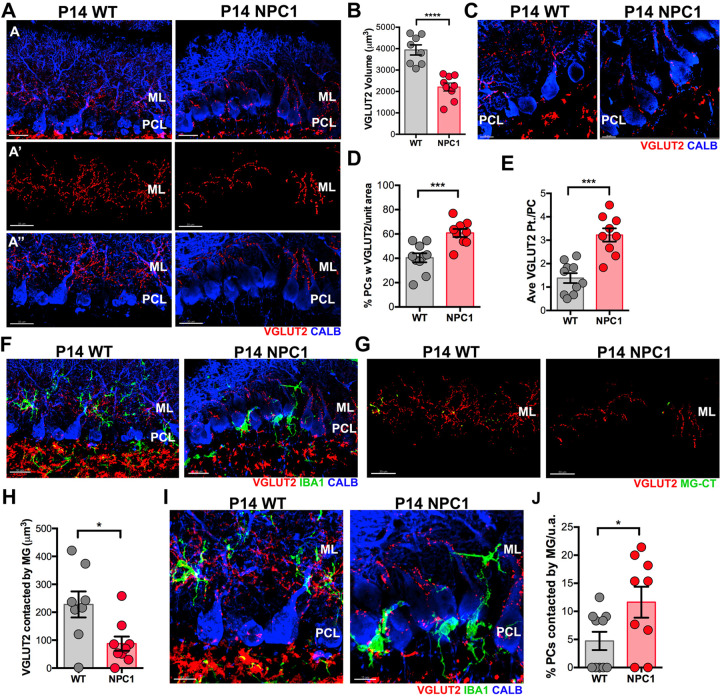
**NPC1 deficiency disrupts CF synapse elimination and translocation in *Npc1^nmf164^* mice.** (A) Representative confocal images from P14 WT and *Npc1^nmf164^* mice showing CALB^+^ PCs innervated by VGLUT2^+^ inputs from CF at the ML. (A′) 3D surface rendered images in which only VGLUT2^+^ inputs in the ML from WT and *Npc1^nmf164^* mice were selected (VGLUT2^+^ inputs in PCL were not included). (A″) Overlap of segregated VGLUT2^+^ inputs (from A′) shown with CALB^+^ PCs to illustrate how only dendritic VGLUT2 was segregated for quantitative analysis. Images of the P14 WT and P14 *Npc1^nmf164^* are from littermate mice. (B) Quantitative analysis of dendritic VGLUT2^+^ inputs at the ML. (C) Representative *z*-section of confocal images showing VGLUT2^+^ inputs in the PCL region innervating CALB^+^ PC somas. (D) Percentage of PC somas containing VGLUT2^+^ inputs. (E) Average of VGLUT2 puncta (Pt) per PC soma. (F) Confocal images from P14 WT and *Npc1^nmf164^* mice showing the distribution and interaction of IBA1^+^ microglia with CALB^+^ PCs innervated by VGLUT2^+^ inputs from CF. (G) Images showing segregated VGLUT2^+^ inputs from the ML contacted or engulfed by microglia (MG-CT, microglia contacts). (H) Quantitative analysis of the volume of VGLUT2^+^ inputs in the ML (innervating dendrites only) contacted by microglia. (I) High magnification images from P14 WT and *Npc1^nmf164^* mice showing the increased interaction of IBA1^+^ microglia with CALB^+^ PC somas innervated by CF VGLUT2^+^ inputs in *Npc1^nmf164^* mice. (J) Quantitative analysis of the percentage of CALB^+^ PC somas contacted by microglia shows a significant increase in *Npc1^nmf164^* mice. Data are presented as mean± s.e.m., WT *n*=8 images from 4 mice, *Npc1^nmf164^ n*=9 images from 4 mice. *P<0.05, ***P<0.001, ****P<0.0001. u.a., unit area. Scale bars: 30 µm (A,F,G); 15 µm (C,I).

During the first 3 weeks after birth, PCs develop their dendritic tree and establish synaptic connections with two major excitatory inputs in the ML: a single climbing fiber (CF) from the inferior olive nuclei and parallel fibers (PFs) from cerebellar granule cells. In the first week, multiple immature synapses with PCs are formed; however, mature and functional synapses are established after the completion of three different periods of synaptic refinement: the early (P8-P11) and late (P12-P17) phases of CF synapse elimination, and the PF synapse elimination phase (P15-P30) (Hashimoto and Kano, 2013; Watanabe and Kano, 2011). During the late phase of CF refinement, the excess of presynaptic terminals is pruned while the terminals of the winning CF are translocating from the PC soma to the proximal regions of PC dendrites (Hashimoto and Kano, 2013; Watanabe and Kano, 2011). The role of microglia during this phase of CF synaptic refinement is not completely understood; however, recent studies have shown that CF synapse elimination is impaired in mouse cerebella depleted of microglial cells (Kana et al., 2019; Nakayama et al., 2018) suggesting a key role of microglia in CF synapse elimination. When we examined P14 cerebella from WT and *Npc1^nmf164^* mice, we found that the volume of CF VGLUT2 (SLC17A6)^+^ presynaptic inputs in the proximal region of CALB^+^ PC dendrites was significantly reduced in *Npc1^nmf164^* mice compared with WT mice (Fig. 8A-B), as previously reported by others (Caporali et al., 2016). However, we also noticed differences in the distribution of VGLUT2^+^ inputs between WT and *Npc1^nmf164^* mice (Fig. 8A-B). In fact, a higher percentage of CALB^+^ PC somas in *Npc1^nmf164^* mice contained VGLUT2^+^ inputs, and VGLUT2 puncta/soma were observed in higher numbers than in WT mice (Fig. 8C-E). Interestingly, significantly more VGLUT2^+^ inputs in the proximal region of CALB^+^ PC dendrites of P14 WT mice were contacted by IBA1^+^ microglia than in the *Npc1^nmf164^* mice (Fig. 8F-H). However, a significantly larger percentage of CALB^+^ PC somas in *Npc1^nmf164^* mice were contacted by IBA1^+^ microglia (Fig. 8I,J), suggesting a possible link between the excess of CF synaptic inputs in PC somas and the increased interaction of microglia with this region of the PC.

**Fig. 9.**
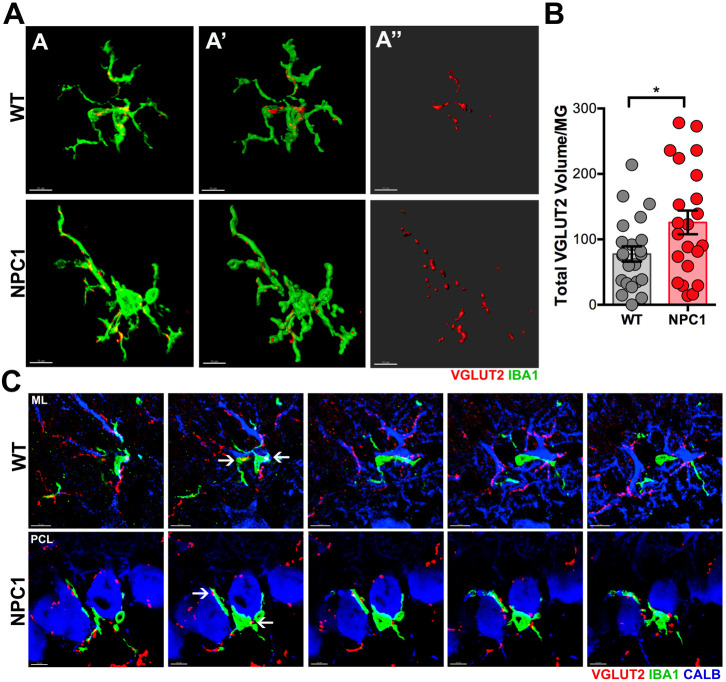
**Increased engulfment of CF presynaptic inputs by microglia in P14 *Npc1^nmf164^* mice.** (A,A′) Confocal images (A) and 3D surface renderings (A′) showing segregated IBA1^+^ microglial cells from P14 WT and *Npc1^nmf164^* mice contacting or engulfing VGLUT2^+^ inputs. (A‴) 3D surface renderings showing only the contacted or engulfed VGLUT2^+^ inputs. (B) Quantitative analysis of the total volume of contacted or engulfed VGLUT2^+^ inputs in P14 IBA1^+^ microglia. (C) High magnification serial sections from the confocal *z*-stack shown in A revealing VGLUT2^+^ inputs innervating CALB^+^ dendrites (WT) or PC somas (*Npc1^nmf164^*) contacted or engulfed by IBA1^+^ microglia at P14. Data are presented as mean±s.e.m., WT *n*=21 cells from 4 mice, *Npc1^nmf164^ n*=22 cells from 4 mice. *P<0.05. Scale bars: 10 µm (A,C).

Because previous studies in other regions of the brain have shown that microglia actively engulf and phagocytose presynaptic terminals during developmental pruning (Gunner et al., 2019; Schafer et al., 2012; Tremblay et al., 2010), and P14 cerebellar microglia were evidently phagocytic as they contained high levels of CD68^+^ phagosomes (Fig. 7G), we analyzed the interaction of individual microglial cells with VGLUT2^+^ presynaptic inputs. To assess whether IBA1^+^ microglia are contacting or engulfing VGLUT2^+^ inputs in WT and *Npc1^nmf164^* mice, confocal microscopy and 3D surface rendering analysis (Imaris) were used in P14 cerebella. At this stage of postnatal development (P14, late phase of CF synapse elimination), WT microglia were actively contacting and engulfing VGLUT2^+^ inputs in the ML (Fig. 9A-A″). Quantitative analysis of the total volume of VGLUT2 puncta per microglia at the PCL and ML indicated that *Npc1^nmf164^* microglia contacted or engulfed significantly more VGLUT2^+^ inputs than WT microglia at P14 (Fig. 9A-B). By examining the *z*-stack sequence images of the microglial cell shown in Fig. 8A, the interactions of the IBA1^+^ cell processes with VGLUT2^+^ inputs innervating CALB^+^ PC dendrites can be observed in Fig. 9C. Some VGLUT2^+^ inputs were completely engulfed (arrows) by the IBA1^+^ cell processes, whereas others were only contacted (Fig. 9C), demonstrating that CF presynaptic inputs are contacted or engulfed by microglia during the late phase of CF refinement. In contrast, *z*-stack imaging sequence of the *Npc1^nmf164^* microglial cell presented in Fig. 8A clearly shows interaction of the IBA1^+^ cell with a CALB^+^ PC soma while also contacting or engulfing VGLUT2^+^ inputs that were found abundantly in this region of the PC in P14 *Npc1^nmf164^* mice (Fig. 9C). These results suggest that NPC1 deficiency not only impairs CF synapse formation, but that it also alters the elimination and translocation of CF synapses in addition to the normal interaction and synaptic pruning function of microglia during the postnatal developmental refinement of CF synapses. Overall, our results show severe impairments in cerebellar microglia and synaptic development that precede and may contribute to early behavioral deficits and neurodegeneration in NPC.

## DISCUSSION

In this study, we demonstrate that deficiency of NPC1 affects the postnatal development and function of cerebellar microglia, contributing to profound defects in developmental synaptic pruning and connectivity in the cerebellum. Specifically, we found that lack of NPC1 in mice reduced radial migration, increased proliferation and impaired differentiation of microglial precursors during the first two postnatal weeks. Increased engulfment of pyknotic bodies and CF presynaptic elements was characteristic of *Npc1^nmf164^* differentiating microglia at 2 weeks of age. These developmental deficiencies precede the pathological changes in microglia, behavioral deficits and degeneration of PCs found in young adult mice (Kavetsky et al., 2019), suggesting that developmental defects in the cerebellum could significantly contribute to psychiatric and neurological symptoms in NPC.

Neuroinflammation is a remarkable pathological hallmark in neurodegenerative diseases that contributes to the progression of neuronal dysfunction and degeneration (Perry et al., 2010; Soto and Howell, 2014). It is thought that neuroinflammation is initiated by microglia activation in response to CNS insult or injury. Microglia activation and neuroinflammation occur in NPC1 patients and mouse models of the disease (Cologna et al., 2014; Cougnoux et al., 2018; Erickson and Bernard, 2002; Kavetsky et al., 2019). Importantly, genetic inactivation of microglia in *Npc1*^−/−^ mice significantly reduces NPC symptoms and increases lifespan (Cougnoux et al., 2018), demonstrating a major role of microglia in NPC progression. Recent work in our laboratory demonstrated that changes in morphology and accumulation of phagosomes in microglia are already observed at early stages of the disease when no loss of PC is detected in *Npc1^nmf164^* mice (Kavetsky et al., 2019). Because these early changes were observed in *Npc1^nmf164^* mice at the end of postnatal development, we investigated whether these changes were an early inflammatory response of microglia or simply a consequence of impaired postnatal development. Gene expression analysis of innate immune response genes in P30 and P90 *Npc1^nmf164^* cerebella revealed that the upregulation of these genes was only detected at the P90 stage, when a severe degeneration of PCs is found, suggesting that the early changes observed in microglia may be the result of disrupted postnatal development. Moreover, at this early age, *Npc1^nmf164^* mice present behavioral deficits, such as repetitive behavior, that seem to be associated with the early pathological changes found in the cerebellum (Kavetsky et al., 2019). Similarly, in the human NPC disease, where the classic presentation of the disease often occurs in middle to late childhood, early neurological symptoms associated with cerebellar dysfunction, such as clumsiness, gait disturbances, and eventually ataxia, are observed before the manifestation of other neurological symptoms (Patterson, 1993). These findings suggest that deficiency of NPC1 causes developmental disturbances in the cerebellum that precede neurodegeneration.

Microglial precursors actively proliferate, migrate and undergo morphological changes during normal brain development (Mosser et al., 2017). A substantial portion of the anatomical and functional development of the mouse cerebellum occurs during postnatal development (Butts et al., 2014). Therefore, in early postnatal days, round and ameboid shape microglial precursors accumulate in the developing CbM, where they actively proliferate and migrate radially to the developing cerebellar cortex (Ashwell, 1990; Mosser et al., 2017). As microglia are the cells in the brain with the highest expression of *Npc1* (Bennett et al., 2016), we hypothesized that NPC1 deficiency severely affects the postnatal development of cerebellar microglia in *Npc1^nmf164^* mice. Indeed, our data demonstrated that early radial migration of microglial precursors was reduced or delayed in *Npc1^nmf164^* mice as fewer microglial precursors were found in the IGL in P4 mice. NPC1 deficiency has been previously implicated in the reduced *in vitro* migration and invasion of CHO and fibroblast cells from NPC patients implicating dysfunctional recruitment and function of integrins in focal adhesion during cell migration (Hoque et al., 2015). It is possible that the intrinsic ability of microglial precursors to migrate is affected by the lack of NPC1 and the lysosomal accumulation of cholesterol.

Another important finding in this study was the increased density of microglia in the developing WMR and cerebellar cortex regions of *Npc1^nmf164^* mice. In the normal brain, microglia are highly proliferative during the first two postnatal weeks, particularly in the developing CbM and WMR (Li et al., 2019; Nikodemova et al., 2015). A recent study demonstrated that the density of a subset of microglial precursors named PAM peaks at P7 exclusively in the cerebellar WMR (Li et al., 2019). In our study, we found higher proliferative activity in microglial precursors at P4 in the CbM, followed by a significantly increased number of microglia in the cerebellar WMR and in the PCL/ML of P10 *Npc1^nmf164^* mice. Furthermore, the number of CLEC7A^+^ PAM in the WMR was also increased in *Npc1^nmf164^* mice, suggesting that NPC1 deficiency amplifies the proliferative activity of microglial precursors during highly proliferative stages. An increased number of microglia was still found at P14 and in post-weaning *Npc1^nmf164^* mice (Kavetsky et al., 2019), indicating that the active proliferation of microglial precursors in the WMR leads to a higher number of these cells in the cerebellar cortex. A few CLEC7A^+^ differentiating microglia were observed in *Npc1^nmf164^* mice at P10, but these cells were no longer seen at P14, indicating a possible failure of these cells to rapidly downregulate *Clec7a* expression during their differentiation at P10. Interestingly, CLEC7A is not only a specific marker for PAM, but also for DAM, which are only found in neurodegenerative conditions (Keren-Shaul et al., 2017; Krasemann et al., 2017), including NPC (Cougnoux et al., 2018). We also confirm the reappearance of CLEC7A^+^ microglia during NPC neurodegeneration in P60 *Npc1^nmf164^* cerebella, but at this stage these CLEC7A^+^ DAM are mainly found at the ML where PC dendrites are degenerating. It is speculated that PAM-derived microglia is transcriptionally predisposed to phagocytosis (Li et al., 2019); therefore, the NPC cerebellum possesses a higher density of these type of microglia that is more phagocytic. In fact, decreased levels of MBP in *Npc1^nmf164^* mice (Caporali et al., 2016) correlate with the increased density of PAM, which actively phagocytose OPCs (Li et al., 2019). Future studies targeting PAM in NPC are needed to understand the role of this subset of cells in the early onset and neurodegeneration of this disease.

Developmental genes such as *Clec7a* are reactivated during neurodegenerative conditions by the TREM2/APOE pathway (Krasemann et al., 2017), which does not activate these genes in PAM during development (Li et al., 2019). Recent *in vitro* studies have shown that lysosomal accumulation of cholesterol in NPC1-deficient cells causes constitutive activation of the mTOR pathway, which is involved in cell growth and proliferation (Castellano et al., 2017; Lim et al., 2019). Intriguingly, overactivation of the mTOR pathway in mouse mature microglia *in vivo* leads to less-ramified microglia, increased proliferation and robust phagocytic activity in the absence of an inflammatory response in a mouse model of epilepsy (Zhao et al., 2018). It is highly probable that NPC1 deficiency causes the pathological changes in developmental microglia through the overactivation of the mTOR pathway, as increased proliferation, impaired differentiation and increased phagocytic activity were hallmarks of postnatal *Npc1^nmf164^* microglia. Further studies are warranted to determine the role of the mTOR signaling pathway in NPC microglia pathology.

Microglial cells play an important role in the clearance of apoptotic cells during neuronal developmental death (Ashwell, 1990; Mosser et al., 2017). However, it has also been demonstrated that microglia can induce apoptosis in the neurons they phagocytose (Mosser et al., 2017). In this study, an abundant number of maturing microglia in the ML containing phagocytic cups in both WT and *Npc1^nmf164^* mice were found at the end of the second postnatal week. It was also evident that the number of phagocytic cups and phagocytic cups containing pyknotic bodies was significantly higher in *Npc1^nmf164^* mice than in WT mice. A high content of phagosomes in P14 microglia at the ML confirmed that at this stage of postnatal development microglial cells were engaged in phagocytic activity. Previous work in the developing rat cerebellum found that the density of phagocytic cups peaks around P17 (Perez-Pouchoulen et al., 2015), supporting our findings that microglia are highly phagocytic by the end of the second postnatal week. It is presumed that pyknotic bodies observed at the ML are apoptotic granule precursor cells that were migrating from the ECL into the IGL during postnatal development (Wood et al., 1993). Interestingly, a reduced number of cerebellar granule cells and reductions in cerebellar lobule size at the end of postnatal development have been found in *Npc1^−/−^* mice (Nusca et al., 2014). It is possible that the increased number of phagocytic cups and the engulfed pyknotic bodies in *Npc1^nmf164^* mice are caused by the increased number of noninflammatory microglia in the developing mutant cerebellum, which could also increase the developmental apoptotic death of cells at the ML. Indeed, an increased number of apoptotic cells was found in mice with elevated microglial phagocytic activity due to the constitutive activation of the mTOR pathway in noninflammatory microglia (Zhao et al., 2018), suggesting that phagocytic microglia can induce and increase developmental neuronal apoptosis.

In the cerebellum, developmental CF synapse elimination and refinement occur during the first three postnatal weeks (Kano and Watanabe, 2019). Initially, multiple CFs innervate the soma of PCs; however, the selective synaptic strengthening of a single CF (P3-P7) begins the early phase of synapse elimination of the redundant CFs synapses (P8-P11). Translocation and expansion of the strongest CF to the proximal region of PC dendrites occur along with the second phase of CF synapse elimination (P12-P17), which is also dependent on excitatory synapse formation between PFs and PC dendrites (Kano and Watanabe, 2019). Here, we found that VGLUT2^+^ synaptic inputs from CFs were significantly reduced in the ML of *Npc1^nmf164^* mice at P14, suggesting that NPC1 deficiency affects CF synapse formation. Our results also indicate that translocation of CF synaptic inputs from the PC soma to the proximal region of PCs dendrites was impaired as a higher number of PC somas contained VGLUT2^+^ and a greater number of VGLUT2^+^ puncta per PC soma were found in *Npc1^nmf164^* mice. A previous study found that not only were the glutamatergic CF synaptic inputs reduced in *Npc1^nmf164^* mice, but also the GABAergic (basket/stellate cells) inputs, indicating that deficiency of the NPC1 protein broadly impairs synaptic connectivity in the cerebellum (Caporali et al., 2016). These synaptic defects were also associated with developmental deficits in motor skill acquisition in the *Npc1^nmf164^* mouse.

Importantly, previous studies have demonstrated that microglia have a role in developmental activity-dependent synaptic pruning in the brain (Gunner et al., 2019; Schafer et al., 2012; Tremblay et al., 2010). In fact, microglia engulf and remove intact presynaptic elements during the process of developmental synaptic pruning (Gunner et al., 2019; Schafer et al., 2012; Tremblay et al., 2010). The role of microglia in developmental CF synapse refinement is not completely understood. However, recent studies have shown that genetic or pharmacological depletion of microglia in the cerebellum impairs the early and late stages of CF synapse elimination during postnatal development leading to behavioral and motor deficits (Kana et al., 2019; Nakayama et al., 2018). Furthermore, it is thought that microglia facilitate developmental CF synapse elimination by promoting GABAergic inhibition of PCs (Nakayama et al., 2018). Here, we aimed to determine whether cerebellar microglia engulf CF presynaptic inputs at P14 (late-phase of CF synapse elimination) and whether NPC1 deficiency alters this microglial pruning function. In fact, we found that at P14, microglia were contacting and engulfing VGLUT2^+^ inputs in the ML of WT mice. These results are in accordance with the abundant density of CD68^+^ phagosomes observed in P14 microglia, indicating that microglia are highly phagocytic in the cerebellum at this postnatal age. Interestingly, at this age, the increased density of somatic VGLUT2^+^ in *Npc1^nmf164^* PCs coincided with a higher percentage of PC somas contacted by microglia. Furthermore, P14 microglia contacted and engulfed more VGLUT2^+^ inputs in *Npc1^nmf164^* mice than in WT mice. It is possible that the reduced elimination and translocation of VGLUT2^+^ inputs in *Npc1^nmf164^* PC somas could be the consequence of decreased GABAergic stimulation to PCs (Caporali et al., 2016), which is also modulated by microglia (Nakayama et al., 2018). Also, it is thought that microglia preferentially engulf and remove presynaptic inputs with decreased activity (Gunner et al., 2019; Schafer et al., 2012; Tremblay et al., 2010), which could explain why a higher number of VGLUT2^+^ inputs are contacted or engulfed by microglia in *Npc1^nmf164^* mice. Disrupted presynaptic terminals in NPC can predispose neurons to early neurodegeneration, as demonstrated in a mouse model of the lysosomal storage disease mucopolysaccharidosis type IIIA, in which restoration of presynaptic function delayed neurodegeneration (Sambri et al., 2017). Current work in our laboratory is investigating whether this phagocytic activity of NPC microglia affects other synaptic refinement and remodeling programs in PCs. Overall, our data demonstrate that deficiency of NPC1 affects microglia and synapse development during the postnatal development of the cerebellum, leading to behavioral deficits and predisposing PCs to neurodegeneration.

## MATERIALS AND METHODS

### Animals

All experiments involving mice were conducted in accordance with policies and procedures described in the Guide for the Care and Use of Laboratory Animals of the National Institutes of Health and were approved by the Animal Care and Use Committees at the Rowan University School of Osteopathic Medicine. The C57BL/6J-*Npc1^nmf164^*/J mouse strain (Jax stock number 004817) was provided by Dr Robert Burgess at The Jackson Laboratory. *Npc1^nmf164^* heterozygous mice were bred and housed in a 12/12-h light/dark cycle to generate both WT and *Npc1^nmf164^* homozygous mutant mice. Both males and females were used in this study, at a ratio of 2:2 when four mice were used.

### Mouse perfusion and tissue preparation

Mice were euthanized with CO_2_ and transcardially perfused with 1× PBS followed by 4% paraformaldehyde. After perfusion, mice were decapitated and their brains were carefully dissected and fixed by immersion in 4% paraformaldehyde overnight. After fixation, brains were rinsed in 1× PBS, immersed in 30% sucrose/PBS solution overnight at 4°C, frozen in OCT, and cryosectioned at 25 μm or 50 μm (floating sections).

### Immunohistochemistry

For immunostaining, brain sections on slides (25 μm) or as floating sections (50 μm) were rinsed once in 1× PBT (PBS+1% Triton-X 100) and incubated with primary antibodies diluted with 1× PBT+20% normal donkey serum overnight at 4°C. After incubation with primary antibodies, sections were rinsed three times with 1× PBT for 10 min and incubated for 2 h in the corresponding secondary antibodies (1:800, Jackson ImmunoResearch or Invitrogen). Tissue was then washed three times with 1× PBT for 10-15 min, incubated with DAPI and mounted in Poly aquamount (Polysciences). The following primary antibodies were used: rabbit anti-IBA1 (1:200, Wako, 019-19741), rat anti-KI67 (1:200, SolA15, Thermo Fisher, 14-5698-82), rat anti-CLEC7A (1:50, 2A11, Bio-Rad, MCA2289), mouse anti-CALB (calbindin, 1:200, Sigma-Aldrich, C9848), rat anti-CD68 (1:200, Bio-Rad, MCA1957), *Lycopersicon esculentum* (Tomato)-Lectin (1:200, Sigma-Aldrich, L0401) and guinea-pig anti-VGLUT2 (1:800, Synaptic Systems, 135 404). The following secondary antibodies were used: Alexa Fluor 647 Streptavidin (ImmunoResearch Laboratories, 016-600-084), Alexa Fluor 568 donkey anti-rabbit (Thermo Fisher, A10042), Alexa Fluor 488 donkey anti-rat (Jackson ImmunoResearch Laboratories, 712-545-150), Alexa Fluor 647 donkey anti-mouse (Jackson ImmunoResearch Laboratories, 715-605-150), Alexa Fluor 594 donkey anti-guinea pig (Jackson ImmunoResearch Laboratories, 706-585-148) and Alexa Fluor 488 donkey anti-rabbit (Jackson ImmunoResearch Laboratories, 711-545-152).

### Microscopy image analysis

To keep consistency between samples, imaging and quantitative analyses to determine changes in the number of IBA1^+^ cells and IBA1^+^/KI-67^+^ cells were performed in the first four anterior cerebellar lobules (I-IV). For quantification of IBA1^+^ and IBA1^+^/KI67^+^ cells in the developing cerebellum, four images (one per lobule) were taken from two cerebellar cryosections for each mouse (eight images per mouse) with an inverted Leica DMi8 fluorescent microscope. For the WMR, one image per section (two sections) were taken using a Kyence microscope. The imaged regions were randomly selected and investigators were blinded to the genotype. Once the images were taken, a box of 250×350 pixels (cerebellar cortex) or 350×450 pixels (CbM) was used to crop the images (one or two boxes per image), so that the area used for the cell counting was consistent between images/animals, and included IGL, PCL/ML, EGL and PS in P4 mice, PCL/ML, EGL and PS in P10 mice, and PCL/ML, EGL and PS in P14 mice. The cropped images were manually counted using the cell counter plugin from the ImageJ (1.47 d) software. For quantification of lectin^+^ IGL capillaries total length, a region of 400×300 pixels was cropped and the Simple Neurite Tracer from ImageJ was used to trace the capillaries and obtain the length measure of every capillary in the image. For quantification of CLEC7A/IBA1 area and MBP intensity, two images per section were taken using a Kyence microscope. The CLEC7A/IBA1 and MBP immunostained area to be measured was selected by threshold and measured by the Analyze plugin of ImageJ. Investigators were blind to the genotype of the tissue while counting the cells or immunostained areas.

For 3D image reconstructions and analyses, three sagittal 50 μm cerebellar sections were immunostained by free-floating immunohistochemistry. All the images analyzed by the Bitplane Imaris software were acquired using a Nikon A1R Confocal System equipped with Live Cell 6 Laser Line and Resonant Dual Scanner. Confocal image stacks were acquired using a 40× objective lens with a 1 μm interval through a 50 μm *z*-depth of the tissue. Three confocal images per mouse were taken from the first three lobes (one per lobe), in the CbM (P4), in the WMR (P10) and in the cerebellar cortex (P10 and P14). To quantify microglial precursors in the WMR of P10 mice, a box of 500×500 μm was used and Imaris surface rendering tool was used to calculate the volume of IBA1^+^ cells and colocalization of IBA1^+^ and KI67^+^ cells inside the box. The quantification of microglia with phagocytic cups and the number of phagocytic cups were quantified manually in 40× confocal images of the ML in P14 cerebella using the cell counter plugin from ImageJ. Two to three images per mouse (*n*=4) were used for the quantifications in confocal images.

Quantitative analysis of 3D microglia morphology was performed using the Surface rendering tool for cell volume and the Filament Tracer for processes volume and ramification; both tools are part of the Bitplane Imaris software. Confocal *z*-stack images of ∼50 μm were taken and twenty IBA1^+^ (five or six per mouse, *n*=4 mice) were segregated using 3D surface rendering to be used for the Filament Tracer tool9, which determines the length, volume and ramification of processes. The 3D surface rendering was also used to segregate IBA1^+^ microglia and to quantify CD68^+^ phagosomes inside microglia, or VGLUT2^+^ synaptic terminals contacted or engulfed by microglia, by using the ‘Mask all’ tool, which creates a new channel of the immunostained areas that are inside the created surface (in this case IBA1 surface), clearing all the fluorescence that is not found overlapping or contacting the rendering surface. The sum of the CD68 or VGLUT2 volume contacted or inside the IBA1 surface was calculated and provided by the software and used for the data analysis presented here. The quantification of VGLUT2 volume in the ML of P14 mice was performed by cropping the ML region (300 μm height×400 μm wide) in 40× confocal images and creating a 3D surface rendering that was used to obtain the sum of the volume of all the VGLUT2^+^ inputs inside the cropped image. To quantify the volume of VGLUT2^+^ inputs contacted or engulfed by microglia in the ML, the ‘Mask all’ tool, which creates a new channel of the IBA1 immunostained area that are in contact or inside the created surface (in this case the VGLUT2 surface), was used, then a new surface was created for the IBA1/VGLUT2 overlapping inputs and the calculated volume sum values were collected. The percentage of CALB^+^ PCs with VGLUT2^+^ inputs and the number of VGLUT2^+^ inputs per cell were quantified manually in 1 μm *z*-sections from 40× confocal images using the cell counter plugin from the ImageJ (1.47 d) software. Two or three images per mouse (*n*=4) were used for these quantifications.

### Quantitative real-time polymerase chain reaction (PCR) array

To measure gene expression changes in mouse innate immune response genes in the cerebellum of WT and *Npc1^nmf164^* mice, real-time PCR was carried out. Cerebella from P30 WT (*n*=4), P30 *Npc1^nmf164^* (*n*=4) and P90 *Npc1^nmf164^* mice (*n*=3) were collected after mice were perfused with 1× PBS and treated overnight in RNAlater (Thermo Fisher, AM7020) for long-term storage. For RNA extraction, 30 mg of cerebellum from each mouse was used and total cellular RNA was extracted and purified from each individual tissue according to the TRIzol Plus RNA Purification Kit (Thermo Fisher, 12183555) manufacturer protocol; RNA concentration and purity were determined using the Qubit 2.0 Fluorometer using an RNA quantification kit (Invitrogen, Q10210). RNA (1 µg) was reverse transcribed to cDNA using the High-Capacity cDNA Reverse Transcription Kit (Thermo Fisher, 4368814). Real-time quantitative PCR was performed using the 96-Well TaqMan Array Mouse Immune Response (Thermo Fisher, 4418724) according to the manufacturer's protocol and one PCR array plate per mouse was used. Briefly, cDNA samples were diluted appropriately, 540 µl cDNA template was added to 540 µl of 2× real-time quantitative reaction mixture (TaqMan Fast Advanced Master Mix, Thermo Fisher), and 10 µl of reaction liquid plus cDNA were added to each well of the PCR array, containing gene-specific primers. Conditions for the real-time quantitative PCR reaction were as follows: UNG incubation 50°C for 2 min, enzyme activation 95°C for 20 s, 40 amplification cycles of denaturing at 95°C for 3 s, annealing/extension at 60°C for 30 s, followed by acquisition of fluorescence signal. Data analysis is based on the ΔΔCt method with normalization of raw signal data to housekeeping genes incorporated on the TaqMan Array Mouse qPCR plate.

### Statistical analysis

Data were analyzed using GraphPad Prism software. Significance was calculated using unpaired *t*-tests for comparisons between two groups. *P*-values are provided as stated by GraphPad Prism software and *P*-values less than 0.05 were considered significant.

## Supplementary Material

Supplementary information

Reviewer comments
