# Supplementary material for: NPC1 deficiency impairs cerebellar postnatal development of microglia and climbing fiber refinement in a mouse model of Niemann–Pick disease type C
Source: Development. 2020 Aug 3;147(21):dev189019. doi: 10.1242/dev.189019 (PMC7420841; doi:10.1242/dev.189019)
Supplement: Supplementary information [file develop-147-189019-s1.pdf]

## Supplementary Information

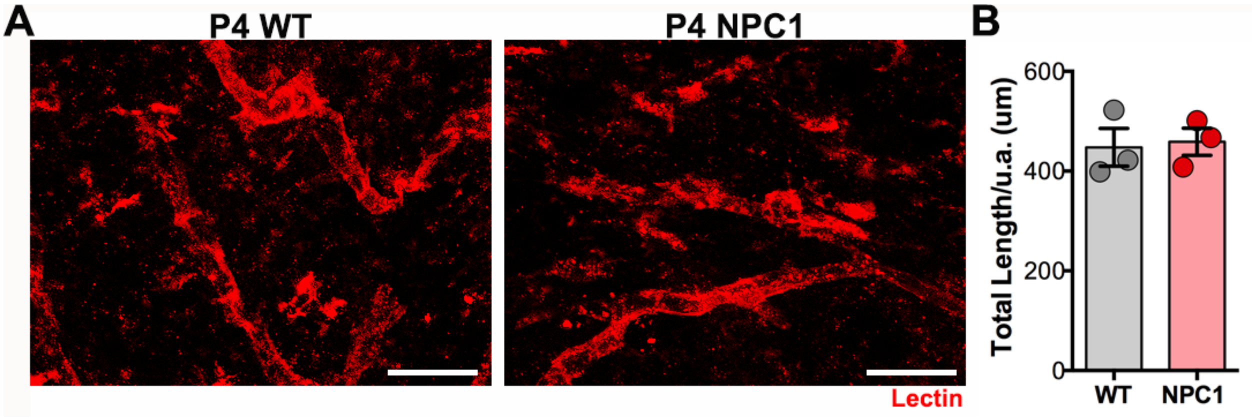


**Fig. S1.** Capillary density in the cerebellar WMR is similar between WT and *Npc1*$^{nmf164}$ mice at P4. A) Sample images showing Lectin$^{+}$ capillaries in the WMR of P4 WT and *Npc1*$^{nmf164}$ mice. B) Quantitative analysis of the total length of Lectin$^{+}$ capillaries in the WMR shows no differences between P4 WT and *Npc1*$^{nmf164}$ mice. Data are presented as mean ± SEM (B) n= 3 mice. Scale bars: (A) 20μm.

Development • Supplementary information

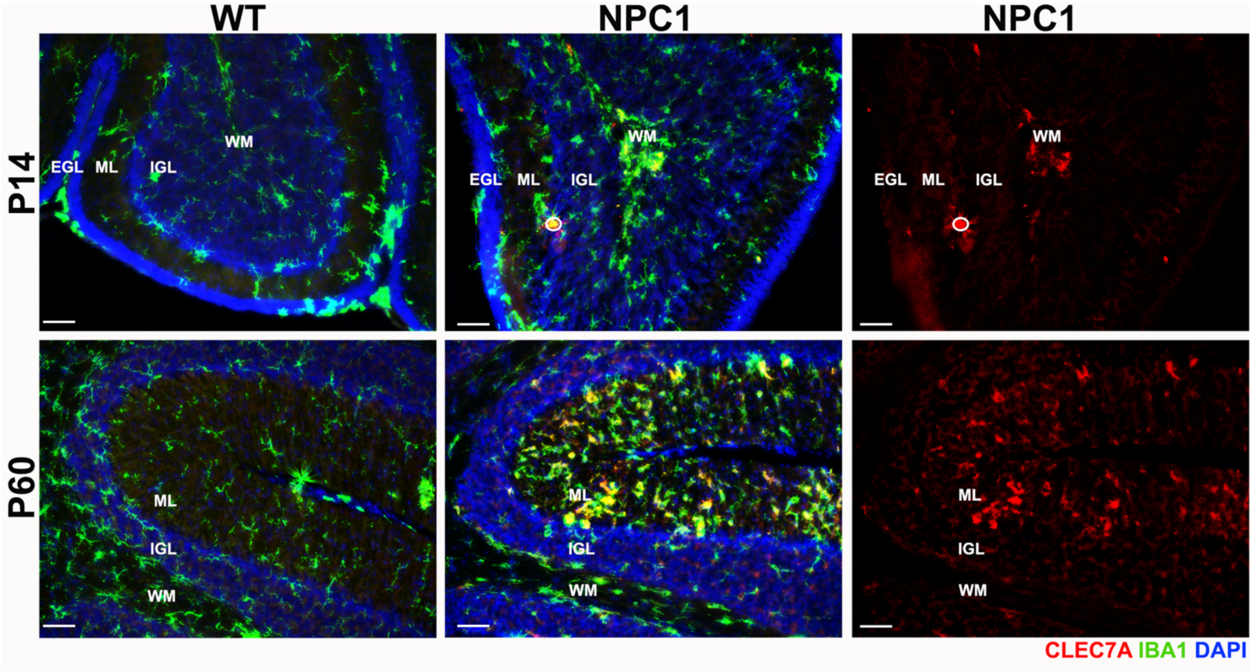


**Fig. S2.** Reappearance of CLEC7A in microglia is observed at P60 when PC degeneration and inflammatory activation of microglia are occurring. At P14, CLEC7A$^+$ microglia were barely detected in the cerebellar white matter (WM) region (or any other region) in WT and *Npc1$^{nmf164}$* mice. At P60, the appearance of CLEC7A$^+$ microglia is evident primarily at the ML where PC dendrite degeneration is occurring. White circle is indicating an artifact. Scale bars: (A) 50μm
