# Supplementary material for: NPC1 deficiency impairs cerebellar postnatal development of microglia and climbing fiber refinement in a mouse model of Niemann–Pick disease type C
Source: Development. 2020 Aug 3;147(21):dev189019. doi: 10.1242/dev.189019 (PMC7420841; doi:10.1242/dev.189019)
Supplement: Reviewer comments [file develop-147-189019_review_history.pdf]

# NPC1 deficiency impairs cerebellar postnatal development of microglia and climbing fiber refinement in a mouse model of Niemann-Pick disease type C.

Bridget R. Boyle, Sierra E. Melli, Ruth S. Altreche, Zachary M. Padron, Fawad A. K. Yousufzai, Sarah Kim, Mariella D. Vasquez, Dawn M. Carone, Benjamin R. Carone and Ileana Soto

DOI: 10.1242/dev.189019

**Editor**: Francois Guillemot

**Review timeline**

Original submission: 14 February 2020
Editorial decision: 6 April 2020
First revision received: 2 June 2020
Accepted: 22 June 2020

---

**Original submission**

First decision letter

MS ID#: DEVELOP/2020/189019

MS TITLE: NPC1 deficiency impairs cerebellar postnatal microglia development and climbing fiber refinement in a mouse model of Niemann-Pick Type C disease.

AUTHORS: Bridget R Boyle, Sierra E Melli, Ruth S Altreche, Zachary M Padron, Fawad A. K. Yousufzai, Sarah Kim, Mariella D Vasquez, Dawn M Carone, Benjamin R Carone, and Ileana Soto

I have now received the reports of two referees on your manuscript and I have reached a decision. The referees' comments are appended below, or you can access them online: please go to BenchPress and click on the 'Manuscripts with Decisions' queue in the Author Area.

As you will see, the referees express great interest in your work,but they also have some significant criticisms and recommend a substantial revision of your manuscript before we can consider publication. If you are able to revise the manuscript along the lines suggested, which may involve further experiments, I will be happy receive a revised version of the manuscript. Your revised paper will be re-reviewed by one or more of the original referees, and acceptance of your manuscript will depend on your addressing satisfactorily the reviewers' major concerns. Please also note that Development will normally permit only one round of major revision.

We are aware that you may currently be unable to access the lab to undertake experimental revisions. If it would be helpful, we encourage you to contact us to discuss your revision in greater detail. Please send us a point-by-point response indicating where you are able to address concerns raised (either experimentally or by changes to the text) and where you will not be able to do so within the normal timeframe of a revision. We will then provide further guidance. Please also note that we are happy to extend revision timeframes as necessary.

Please attend to all of the reviewers' comments and ensure that you clearly highlight all changes made in the revised manuscript. Please avoid using 'Tracked changes' in Word files as these are lost in PDF conversion. I should be grateful if you would also provide a point-by-point response detailing

© 2020. Published by The Company of Biologists under the terms of the Creative Commons Attribution License (https://creativecommons.org/licenses/by/4.0/).

how you have dealt with the points raised by the reviewers in the 'Response to Reviewers' box. If you do not agree with any of their criticisms or suggestions please explain clearly why this is so.

Reviewer 1

*Advance summary and potential significance to field*

Boyle et al investigate early postnatal microglia development and migration in the cerebellum of Npc1nmf164 mice and show that NPC1 deficiency impairs microglia differentiation and ramification during postnatal development, with consequence on synaptic development that precede and may contribute to early behavioral deficits and neurodegeneration in NPC. They first rule out the presence of an immune or inflammatory response in Npc1nmf164 mice at early age, and show that NPC1 deficiency affects the ability of microglia precursors to migrate radially during postnatal cerebellar development. They also show an increased proliferation of white matter microglia precursors in Npc1nmf164 mice that lead to an increased density of microglia in the cerebellar cortex region. They further show that microglia in Npc1nmf164 mice are less ramified and have shorter processes. Finally, they show that NPC1 deficiency alters microglial function including phagocytic activity and the distribution of phagosomes in the developing cerebellar microglia, as well as climbing fiber synapse elimination.

*Comments for the author*

Overall this is an interesting study that however remains fundamentally descriptive and correlative in nature.
The major limitation is that the authors used a model where the mutation is not restricted to any specific lineage and especially not to microglia and hence are not able to distinguish intrinsic and extrinsic effects that could impact the phenotype of microglia.
In the Figure 2B, there is no quantification of EGL.
In the Figure 3C, a dramatic reduction of Ki67+ cells is observed in non IBA1+ cells. Is this something reproducible? That suggest as alluded before several non-microglia specific related effects that could have indirect consequences?
The authors observed an increased number of differentiating microglia in Npc1nmf164 mice at P14. Is it embryonic microglia or newly-recruited microglia?

Reviewer 2

*Advance summary and potential significance to field*

In this study Boyle et al. investigate early developmental defects in microglia prior to Purkinje cell degeneration in a model of Niemann-Pick Type C (NPC). This study is a logical extension of prior work by Kavetsky et al., 2019 that revealed alterations to microglial cell density, morphology, and association with dendrites at 4 weeks+, suggesting that early changes to microglia may account for some of the perturbations in Purkinje cells associated modifications to synaptic connectivity, and neurological phenotypes. This concept is supported by Cougnoux et al., 2018 using NPC KO mice where deletion of activated microglia ameliorates some of the neurological deficits and expands lifespan of mice.

To further understand when and how microglia are altered in NPCnmf164 mice, Boyle et al. study the impact of reducing NPC1 during cerebellar development with a focus on early postnatal alterations to microglial cell proliferation, migration, positioning, and maturation.
They further investigate how early alterations to microglia (prior to any detectable signs of neurodegeneration) affect their interaction with maturing climbing fibers as microglia are known to contribute to climbing fiber reorganization during development.

*Comments for the author*

The experimental approaches applied are appropriate and of high quality. However additional analysis is needed to provide more direct evidence of the cellular phenotypes discovered and strengthen the interpretations and overall impact of the results.

© 2020. Published by The Company of Biologists under the terms of the Creative Commons Attribution License (https://creativecommons.org/licenses/by/4.0/).

Major Points:
1. Figure 2C (related to the manuscript text on Page 7). As a reference point, it would be helpful to quantify the vascular elaboration/density at P4 in WT and NPCnmf164 mice. If there is no significant change then this would provide strong evidence that early vascular changes are unlikely a contributing factor to the defects in microglial positioning.
2. To strengthen the interpretation that there is increased microglial cell proliferation prior to P10, it is important to actually show this, for example, by quantifying KI67 levels at P7 at the peak of microglial proliferation. This will better tie together the analysis at P4 and P10 and provide direct evidence that proliferation is increased and likely accounts for the higher microglial cell density in NPCnmf164 mice.
3. The results suggest that developing microglia in NPCnmf164 mice have increased phagocytic behavior. Thus microglia could be in a temporary/transient activated state during development. This possibility complicates the interpretation that microglial alterations are being caused by lack of differentiation and ramification. It would be helpful to better understand the possibility of microglia activation by looking for upregulation of other markers for activated microglia (i.e. MHC II, CD11b, CD45, etc.). Running the PCR array at P10 from Figure 1B would also help determine the possibility of early activation of microglia that subsides by P30.
4. The authors show that microglia have altered interactions with climbing fibers inputs onto Purkinje cells. However, as climbing fiber development is sensitive to a variety of cerebellar perturbations, it would be important to know if the alteration seen in NPCnmf164 mice is specific to climbing fibers or is reflective of a more generalized microglial cell process. This could be accomplished by examining microglial cell interactions with parallel fiber terminals (vGlut1+) and inhibitory terminals (vGat+).

Minor Points:
1.Page 6, related to Figure 1, it would be useful to mention some of the genes that are altered at P90 but not at P30 and indicate the log-fold change. Also, demarcate which genes are significantly changed in Figure 1B.
2. Page 7, related to Figure 2A, add, "cerebral" to read "cerebral cortex" and to distinguish from "cerebellar cortex"
3. Figure 2A panel. Indicate which part of the cerebellum is shown.

---

**First revision**

Author response to reviewers' comments

Responses to Reviewers' comments

Reviewer 1 Comments for the author

Overall this is an interesting study that however remains fundamentally descriptive and correlative in nature. The major limitation is that the authors used a model where the mutation is not restricted to any specific lineage and especially not to microglia and hence are not able to distinguish intrinsic and extrinsic effects that could impact the phenotype of microglia.

Overall response: We appreciate the reviewer's valuable comments. We agree with Reviewer 1's opinion that the use of a mouse with the Npc1 mutation specifically in microglia could distinguish intrinsic from extrinsic effects of the mutation and we hope to perform those experiments in the near future. However, the results of this current study are showing how the deficiency of NPC1 can affect the brain development of NPC patients. Thank you for taking the time to perform such a careful review of our manuscript. We also want to add that we have included in the results section new data that support the impact of Npc1 deficiency on microglia precursors proliferation. We are hoping the newly added data will strengthen and complement the results that were already included in the first submission of the manuscript.

© 2020. Published by The Company of Biologists under the terms of the Creative Commons Attribution License (https://creativecommons.org/licenses/by/4.0/).

1. In the Figure 2B, there is no quantification of EGL.

Response 1: The many granule precursor cells in the EGL are very packed, and early in the process of quantification we noticed that microglia were either over (PS) or below (ML) the EGL, so we decided to quantify those cells as part of the PS (pia surface) or ML (molecular layer). Other reports have also determined that the EGL is devoid of microglia. I have included the references in the following sentence in the results section (highlighted on blue):

"At this early postnatal stage, very few IBA1+ microglia precursors have reached the PCL, and the EGL is completely devoid of them as reported by others (Cuadros et al., 1997; Nakayama et al., 2018)." Page 7.

2. In the Figure 3C, a dramatic reduction of Ki67+ cells is observed in non IBA1+ cells. Is this something reproducible? That suggest as alluded before several non-microglia specific related effects that could have indirect consequences?

Response 2: Thanks for the observation. I examined all the tissue that we analyzed in this study and noticed that it was a variability of the staining which happened in both wild type and Npc1 mutant tissues. I observed that in WT and NPC1 cerebellar slices some regions have fainter labeling than others but it was more a technical issue than a biological result since the variability did not follow any specific pattern and it was not associated with the genotype.

3. The authors observed an increased number of differentiating microglia in Npc1nmf164 mice at P14. Is it embryonic microglia or newly-recruited microglia?

Response 3: This is a very important question and we hope that the new data we have included in this revised manuscript show that is the result of increased proliferation of microglia precursors (embryonic) in the cerebellar medulla and white matter regions of the cerebellar folia. During cerebellar postnatal development, microglia precursors, which are embryonic cells, populate first the cerebellar medulla, proliferate in that region, and migrate towards the cerebellar cortex following axonal tracts in the folia that eventually will become the white matter region (WMR) of the cerebellum. These microglia precursors in the WMR proliferate during the first postnatal days, peaking at 7 days, and significantly decreasing after P7. We have included new data that shows a higher number of Ki67+ microglia precursors in the cerebellar medulla of P4 NPC1 mice. We also added data that show that the number of a type of microglia precursor in the WMR known as "proliferative-region-associated microglia" (PAM), which is CLEC7A+, is significantly increased in the WMR of NPC1 mice at P10. It is known that the proliferation of PAM peaks at P7 and significantly decreases after this age, supporting the higher proliferation of embryonically derived microglia in NPC1. Those precursor cells in the WMR eventually differentiate and migrate into the cerebellar cortex (ML). Since we did not find high proliferative activity in the ML at P10 and P14, we concluded that the increased number of microglia in P14 mice is the result of the increased proliferation of microglia precursors in the WMR. The new data is presented in the new figure #4, and the description of these results is highlighted in blue in the revised manuscript (pages 9-10).

Reviewer 2 Comments for the author

The experimental approaches applied are appropriate and of high quality. However, additional analysis is needed to provide more direct evidence of the cellular phenotypes discovered and strengthen the interpretations and overall impact of the results.

Overall response: We appreciate the reviewer's comments and are particularly grateful for the insightful comments and suggestions that are detailed below. Thank you for taking the time to perform such a careful review of our manuscript and for the suggested analyses to strengthen the findings of this study. As we mentioned before to Reviewer #1, we have included in the results section new data that support the impact of Npc1 deficiency on microglia precursors proliferation (Figure 4). We hope this new data will strengthen and complement the results that were already included in the first submission of the manuscript.

© 2020. Published by The Company of Biologists under the terms of the Creative Commons Attribution License (https://creativecommons.org/licenses/by/4.0/).

Major Points:

1. Figure 2C (related to the manuscript text on Page 7). As a reference point, it would be helpful to quantify the vascular elaboration/density at P4 in WT and NPCnmf164 mice. If there is no significant change, then this would provide strong evidence that early vascular changes are unlikely a contributing factor to the defects in microglial positioning.

Response 1: We did the quantification of the total length of capillaries in the white matter region (WMR) per unit area and found no differences between WT and NPC1 mice. These data are included in Supplementary Figure 1. We have highlighted in blue the addition of "Fig. S1" to the description of the results on page 7.

2. To strengthen the interpretation that there is increased microglial cell proliferation prior to P10, it is important to actually show this, for example, by quantifying KI67 levels at P7 at the peak of microglial proliferation. This will better tie together the analysis at P4 and P10 and provide direct evidence that proliferation is increased and likely accounts for the higher microglial cell density in NPCnmf164 mice.

Response 2: We agree with the reviewer's comment and since we currently do not have P7 tissue or mice to dissect the tissue due to the pandemic, we realized that we did not quantify the proliferative activity of microglia precursors in the P4 cerebellar medulla, where the proliferative activity of these cells start even before P1. Ashwell (1990, cited in the manuscript) reported that the number of microglia precursors in the cerebellar medulla starts to increase after birth, peaks at P7 and is significantly decreased by P9, indicating that the proliferative activity mainly occurs before P7 and that the decrease of microglia precursors in that region is due to the migration and maturation of these cells. We quantified KI67+ microglia precursors in the cerebellar medulla at P4 and found a higher number of these cells in the NPC1 mice. In addition, we used the CLEC7A antibody to specifically immunolabel "proliferative-region-associated microglia" (PAM) which are specifically found in the WMR, and their proliferative activity peaks at P7. Our analysis shows that this subpopulation of microglia precursors is increased in NPC1 mice when compared to WT, supporting that NPC1 deficiency increases microglia precursors proliferation in the WMR leading to a higher number of microglia in the cerebellum at P14. All these data are presented in the new Figure 4, and the description of this figure is highlighted in blue in the manuscript. We are very grateful to the reviewer for their suggestion and excited to include these data in this manuscript to strengthen the results of this study.

3.The results suggest that developing microglia in NPCnmf164 mice have increased phagocytic behavior. Thus, microglia could be in a temporary/transient activated state during development. This possibility complicates the interpretation that microglial alterations are being caused by lack of differentiation and ramification. It would be helpful to better understand the possibility of microglia activation by looking for upregulation of other markers for activated microglia (i.e. MHC II, CD11b, CD45, etc.). Running the PCR array at P10 from Figure 1B would also help determine the possibility of early activation of microglia that subsides by P30.

Response 3: I agree with the reviewer that the interpretation of the data seems complicated due to the possibility of a transient activation. However, during developmental stages is completely normal for microglia to be phagocytic and to play important roles in the clearance of apoptotic cells and the pruning of redundant synapses. Our data suggest that NPC1 microglia are overdoing these functions probably in response to developmental deficits. WT microglia had phagocytic cups at P14, but NPC1 mice had more phagocytic cups, WT microglia engulfed presynaptic terminals contacting PC dendrites, but NPC1 microglia engulfed more and they were located in the PC soma region where these presynaptic terminals were not supposed to be at P14. To test if NPC1 microglia at P14 were "activated" like during inflammatory-degenerative conditions, we used again the detection of the CLEC7A protein, which expression is absent in resting microglia but reactivated in highly phagocytic diseased-associated microglia (DAM, Krasemann et al., 2017), to see if the P14 phagocytic microglia were reactivating the expression of this protein. We found that CLEC7A immunoreactivity reappeared in ML microglia at P60 (a neurodegenerative stage in NPC) in NPC1 mice, presumably in DAMs, but no immunoreactivity was detected in the P14 phagocytic NPC1 microglia at the ML (new Supplemental Figure 2, description highlighted in blue in the results section). These data support that the altered behavior of microglia during development in NPC is

© 2020. Published by The Company of Biologists under the terms of the Creative Commons Attribution License (https://creativecommons.org/licenses/by/4.0/).

not like the immunological or inflammatory response that is observed during neurodegeneration. However, I still think that it is some form of activation since resting microglia is completely ramified with extended and thin processes. The same changes we see in the developmental microglia (morphology, phagocytic activity, proliferation) were observed in adult microglia with constitutive activation of the mTOR pathway, and they referred to these cells as “noninflammatory microglia” (Zhao 2018), I think the NPC1 developmental microglia are noninflammatory (activated) microglia. In the manuscript, we discuss the results of the Zhao study and the relevance of their work on the interpretation of our data.

4.The authors show that microglia have altered interactions with climbing fibers inputs onto Purkinje cells. However, as climbing fiber development is sensitive to a variety of cerebellar perturbations, it would be important to know if the alteration seen in NPCnmf164 mice is specific to climbing fibers or is reflective of a more generalized microglial cell process. This could be accomplished by examining microglial cell interactions with parallel fiber terminals (vGlut1+) and inhibitory terminals (vGat+).

Response 4: This is also a very important point. At the moment of the first submission of this manuscript, we were working on the next study which is focusing on the effects of NPC1 deficiency in the development of Purkinje cells. The Purkinje cell dendrites are remodeled from multiplanar to mono-planar between 18 to 25 days of age, coinciding with the time of the parallel fibers refinement. We have produced data that indicate that NPC1 microglia are also engulfing more VGLUT1 presynaptic terminals at 21 days than WT microglia, so we know that the NPC1 microglia phagocytic behavior is a more generalized process. It has been reported by others that VGLUT1 and VGAT are also decreased in NPC (Carporali et al., 2016, cited in the manuscript). However, if it is ok with the reviewer, we would like to keep that data for the Purkinje cell development/remodeling story, which (before the stay at home orders) we were collecting data that were indicating that NPC1 deficiency significantly alters dendrites development, and a possible role of microglia in dendritic remodeling and PF synapses pruning. I have included the following sentence in the discussion section of the manuscript (page 22):

“Current work in our laboratory is investigating if this phagocytic activity of NPC microglia affects other synaptic refinement and remodeling programs in PCs.”

Minor Points:

1. Page 6, related to Figure 1, it would be useful to mention some of the genes that are altered at P90 but not at P30 and indicate the log-fold change. Also, demarcate which genes are significantly changed in Figure 1B.

Response 1: To improve the presentation of data in Figure 1, we removed all the genes that their log-fold change was not statistically significant. We only include genes that had a log-fold change of 5 or more. The significance of the changes is indicated in the graph and legend. Due to the limit of words required by the journal we could not add a broad explanation about the genes, but we included the following sentence in the results section (page 6):

“As expected, several genes including cytokines (Lif, Tnf, Csf3, Il1a, Ccl2, and Ccl3), endothelial-inflammatory genes (Sele and Vcam1), T-cell associated genes (Gzmb, Cd3e, Cd28, Stat4, PRF1, and Tnfrsf18) and other proinflammatory molecules (C3 and Ptgs2) were significantly increased (>5 fold) in the cerebellum of P90 Npc1nmf164 mice when compared to WT mice (Fig. 1B).”

2. Page 7, related to Figure 2A, add, "cerebral" to read "cerebral cortex" and to distinguish from "cerebellar cortex"

Response 2: Good point. We added cerebral to the figure and the text in the manuscript.

3.Figure 2A panel. Indicate which part of the cerebellum is shown.

Response 3: We have added “Cerebellar Medulla”. I was naming that region before (first submission) white matter region, however, I noticed that at P4 there is no myelination yet, so I saw another publication naming it the cerebellar medulla. This name will also help to distinguish this

© 2020. Published by The Company of Biologists under the terms of the Creative Commons Attribution License (https://creativecommons.org/licenses/by/4.0/).

region from the WMR in the folia, which is also still primitive at P4, but it is more relevant at P10 when myelination is already happening.

---

Second decision letter

MS ID#: DEVELOP/2020/189019

MS TITLE: NPC1 deficiency impairs cerebellar postnatal microglia development and climbing fiber refinement in a mouse model of Niemann-Pick Type C disease.

AUTHORS: Bridget R Boyle, Sierra E Melli, Ruth S Altreche, Zachary M Padron, Fawad A K Yousufzai, Sarah Kim, Mariella D Vasquez, Dawn M Carone, Benjamin R Carone, and Ileana Soto
ARTICLE TYPE: Research Article

I am delighted to tell you that your manuscript has been accepted for publication in Development, pending our standard ethics checks.

Reviewer 1

*Advance summary and potential significance to field*

The authors have adequately addressed most of the points that were raised. However, they did not addressed the origin of the increased number of differentiating microglia in Npc1nmf164 mice at P14 which is an important question.

*Comments for the author*

The authors have adequately addressed most of the points that were raised. However, they did not addressed the origin of the increased number of differentiating microglia in Npc1nmf164 mice at P14 which is an important question.

Reviewer 2

*Advance summary and potential significance to field*

The authors have sufficiently addressed my concerns with the inclusion of new results and manuscript editing.

*Comments for the author*

The authors have sufficiently addressed my concerns.

© 2020. Published by The Company of Biologists under the terms of the Creative Commons Attribution License (https://creativecommons.org/licenses/by/4.0/).
